# Visit‐to‐visit variability of blood pressure and risk of macrovascular and microvascular complications in patients with type 2 diabetes: A Chinese primary‐care cohort study

**DOI:** 10.1111/1753-0407.13331

**Published:** 2022-11-28

**Authors:** Ying Deng, Yin Liu, Shengchao Zhang, Hanbing Yu, Xiaozhou Zeng, Rongrong An, Zhenyuan Chen, Na Sun, Xiaoxv Yin, Yue Dong

**Affiliations:** ^1^ Department of Community Health Management Baoan District Central Hospital Shenzhen People's Republic of China; ^2^ Department of Social Medicine and Health Management, School of Public Health, Tongji Medical College Huazhong University of Science and Technology Wuhan People's Republic of China

**Keywords:** blood pressure, macrovascular complications, microvascular complications, type 2 diabetes mellitus, 血压, 大血管并发症, 微血管并发症, 2型糖尿病

## Abstract

**Background:**

We evaluated the effects of visit‐to‐visit variability of systolic blood pressure (SBP) and diastolic blood pressure (DBP) on macrovascular and microvascular complications among patients with type 2 diabetes.

**Methods:**

A total of 11 043 patients with type 2 diabetes from primary healthcare institutions between January 2010 and June 2020 were included. The visit‐to‐visit blood pressure variability was calculated using three metrics: SD, coefficient of variation (CV), and average real variability (ARV), obtained over a 12‐month measurement period. The associations of visit‐to‐visit blood pressure variability with macrovascular and microvascular complications were evaluated using multivariate‐adjusted Cox proportional hazards models, and hazard ratio (HR) with 95% confidence interval (CI) were reported.

**Results:**

There were 330 macrovascular events and 542 microvascular events. Compared to those for participants with the lowest quartile of SD of SBP and DBP, increased risks were observed in patients with the highest quartile of SD of SBP and DBP for macrovascular complications (SD‐SBP: HR = 1.78, 95% CI: 1.24–2.57; SD‐DBP: HR = 2.20, 95% CI: 1.50–3.25) and microvascular complications (SD‐SBP: HR = 1.85, 95% CI: 1.39–2.46; SD‐DBP: HR = 1.82, 95% CI: 1.36–2.44). CV and ARV of SBP and DBP also had statistically significant associations with macrovascular and microvascular complications. The optimal variability of blood pressure target was SD of SBP <6.45 mm Hg and SD of DBP <4.81 mm Hg.

**Conclusions:**

Visit‐to‐visit blood pressure variability may be a potential predictor for macrovascular and microvascular complications in patients with type 2 diabetes.

## Graphical abstract

**Highlights**

- Evidence on the associations between visit‐to‐visit variability of blood pressure and risks of macrovascular and microvascular complications in patients with type 2 diabetes was insufficient and controversial.
- This study showed that greater variability of blood pressure was related to increased risks of macrovascular and microvascular complications for patients with type 2 diabetes.
- Our findings identified the optimal variability of blood pressure target for patients. Clinical professionals should also remain vigilant about the visit‐to‐visit fluctuation of blood pressure.

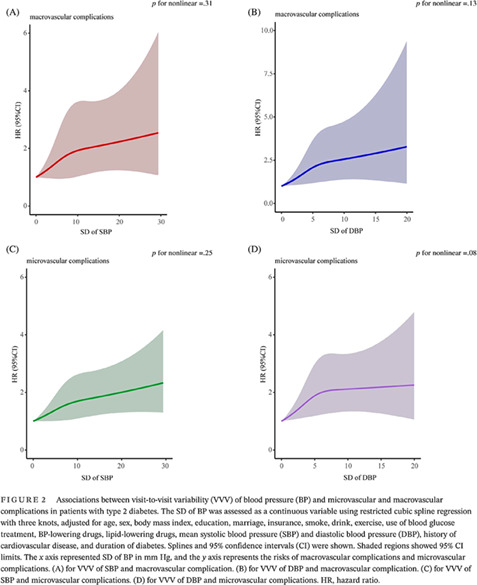

## 摘要

**背景:**

我们评估了在2型糖尿病患者就诊间收缩压(SBP)和舒张压(DBP)的变异性与大血管和微血管并发症的关系。

**方法:**

纳入2010年1月至2020年6月在基层医疗机构就诊的11043例2型糖尿病患者。就诊间的血压变异性使用在12个月的测量期内获得的三个指标计算:标准差(SD), 变异系数(CV)和平均实际变异性(ARV)。用多变量校正的COX比例风险模型评估就诊间的血压变异性与大血管和微血管并发症的关系，并报告95%可信区间(CI)的危险比(HR)。

**结果:**

大血管事件330例，微血管事件542例。与SBP和DBP标准差最低四分位数的受试者相比，SBP和DBP标准差最高四分位数的患者发生大血管并发症(SD‐SBP:HR=1.78，95%CI:1.24‐2.57, SD‐DBP:HR=2.20，95%CI:1.50‐3.25)和微血管并发症(SD‐SBP:HR=1.85，95%CI:1.39‐2.46, SD‐DBP:HR=1.82，95%CI:1.36‐2.44)的风险增加。SBP和DBP的CV和ARV也与大血管和微血管并发症有统计学意义的相关性。最佳血压指标变异性为:SBP标准差<6.45 mm Hg，DBP标准差<4.81 mm Hg。

**结论:**

就诊间的血压变异性可能是2型糖尿病患者大血管和微血管并发症的潜在预测指标。

## 1 INTRODUCTION

Diabetes is a public health problem.^1^, ^2^ Several studies have demonstrated that diabetes is associated with higher risk of cardiovascular diseases (CVD).^3^, ^4^, ^5^ Therefore, effective health management is essential to prevent cardiovascular complications for patients with diabetes. In recent years, many countries have published guidelines on diabetes management, emphasizing the importance of blood pressure (BP) monitoring for patients with type 2 diabetes and advocating maintaining optimal blood pressure to prevent CVD.^6^, ^7^ In addition to absolute BP readings, increasing attention is being paid to the harmful effect of BP variability in patients with type 2 diabetes.^8^

Current studies of visit‐to‐visit blood pressure variability (BPV) were mainly focused on the relationship between visit‐to‐visit variability (VVV) of systolic blood pressure (SBP) and CVD. Several studies have reported that VVV of SBP is a risk factor for CVD in patients with type 2 diabetes,^9^, ^10^, ^11^ and a recent systematic review and meta‐analysis concluded that the VVV of SBP was associated with CVD and cardiovascular mortality for patients with type 2 diabetes.^12^ However, evidence for the association between VVV of diastolic blood pressure (DBP) and CVD was limited.^12^ The results from previous studies were inconsistent, some reporting a nonsignificant relationship,^13^ with others pointing to a significant association.^14^ Furthermore, most of the previous studies were conducted in developed countries, particularly in North American and European regions. There is a paucity of studies on BPV and macrovascular and microvascular complications in patients with type 2 diabetes in China.

The total number of adults (20–79 years) with diabetes in China was up to 140.9 million in 2021, which now has the largest number of adults with diabetes worldwide.^15^ Several diabetes management guidelines have been formulated in China, recommending monitoring BP frequently and controlling BP reasonably.^16^, ^17^ However, associations on BPV and CVD in patients with type 2 diabetes in China were still unclear. Therefore, our study aimed to explore the relationships between BPV and the risks of macrovascular and microvascular complications among patients with type 2 diabetes based on the Health Management Program of Diabetes (HMPD) in China.

## 2 METHODS

This study was approved by the ethics committee of Huazhong University of Science and Technology (Number: 2022S004).

### 2.1 Data resource

Our data were drawn from the medical records of HMPD in Community Health Service Centers from Xixiang subdistrict, Shenzhen, China. A total of 38 community healthcare centers are located at Xixiang subdistrict, covering 33 communities and providing medical service for over 1.5 million residents. According to guidelines of the HMPD, general practitioners in the community healthcare centers carry out health management for patients with diabetes in the catchment, including regular follow‐up evaluations and health examinations. The main procedures of HMPD are conducted as follows. First, general practitioners set up health records for patients with type 2 diabetes, which contain information including demographics (such as age and sex), physical checkup information (such as BP, weight measurement), and disease‐related information (such as duration of diabetes and history of CVD). Patients are then followed up every 3 months and undergo health checkups every year. The quarterly follow‐up evaluations gather information on physical checkups (such as BP, weight measurement), lifestyle characteristics (such as smoking status and drinking habit), treatment modalities of the patients (such as use of antihypertensive drugs, oral antidiabetic drugs or insulin, and lipid‐lowering drugs), and disease status (diagnosed with macrovascular and microvascular complications). Annual health checkups contain diagnosis of diabetes‐related complications (stroke, myocardial infarction, nephropathy, neuropathy, and retinopathy).

### 2.2 Clinical blood pressure measurements and visit‐to‐visit variability of blood pressure

BP measurement period was defined as 12 months. During this period, patients were followed up quarterly, and BP was measured during each follow‐up. One BP value for each patient was recorded during each follow‐up examination. However, we performed multiple BP measurements for each patient. The procedure of obtaining BP value for patients with type 2 diabetes followed standardized guidelines, which were applicable to all general practitioners.^18^ BP was measured after allowing the patients to rest for at least 5 min without any distractions, in a seated position. Two measurements were taken at each visit, with an interval of at least 1 min. If the difference between these two readings did not exceed 5 mm Hg, the average of these two readings was defined as the BP value for this follow‐up evaluation. Otherwise, an additional measurement would be performed, with an interval of 2 min. The average of these three readings was recorded.

SD was used as the main metric of BPV, coefficient of variation (CV) and average real variability (ARV) were used as supplementary metrics. Detailed information for all metrics of BPV was as follows. To recognize the effects of different degrees of BPV, all patients were categorized into four groups based on their SD, CV, and ARV quartiles.

$$\mathrm{SD}=\sqrt{\frac{\sum_{i=1}^{n}{\left(x_{i}-\bar{x}\right)}^{2}}{\left(n-1\right)}}$$

$$\mathrm{CV}=100*\mathrm{SD}/\bar{x}$$

$$\mathrm{ARV}=\frac{1}{n-1}\sum_{i=1}^{n-1}|x_{i+1}-x_{i}|$$

*n* stands for the number of blood pressure measurements. $x_{i}$ stands for the blood pressure value for certain measurement.

### 2.3 Study outcomes

The outcomes of this research consisted of *International Classification of Diseases, Tenth Revision* (ICD‐10) coded macrovascular and microvascular complications, which derived from two sources. First, cardiovascular diseases were diagnosed by physicians based on laboratory examinations, imaging examinations, and clinical symptoms during annual health examinations. The diagnosis results were then uploaded to the medical records. Second, doctors asked patients whether they had been diagnosed with cardiovascular diseases in other medical institutions and uploaded responses to their medical records. Macrovascular complications included stroke (ICD‐10 I60, I61, I63, and I64) and myocardial infarction (ICD‐10 I21). Microvascular complications covered diabetic kidney disease (ICD‐10 E11.2), diabetic retinopathy (ICD‐10 E11.3), and diabetic neuropathy (ICD‐10 E11.4). The end point of outcomes assessment was June 2021.

### 2.4 Study population

**FIGURE 1:**
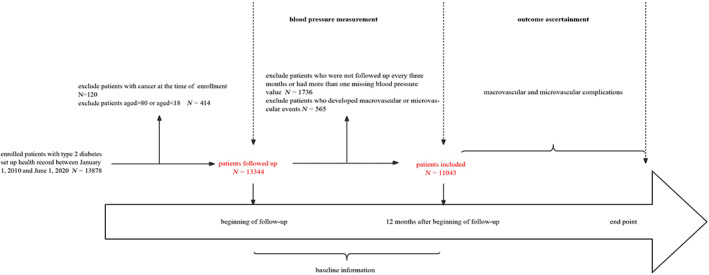
Timeline of the study design: Blood pressure measured at four occasions (3, 6, 9, and 12 months) was used to determine the mean BP and visit‐to‐visit variability of blood pressure. Excluding patients who were aged >80 or aged <18, patients with cancer, patients who experienced macrovascular or microvascular events within 12 months, and missing blood pressure values at any of four occasions, 11 403 patients were eligible for the analysis.

We enrolled 13 878 patients with type 2 diabetes from the HMPD between 1 January 2010 and 1 June 2020. The exclusion criteria were as follows: (1) patients with cancer at the time of enrollment (*N* = 120); (2) patients aged >80 years or < 18 years (*N* = 414); (3) patients who were not followed up every 3 months or had >1 missing BP value during the 12‐month measurement period (*N* = 1736); and (4) patients who developed macrovascular or microvascular complications during the time of 12‐month measurement period (*N* = 565). It is important to note that we did not exclude patients with history of CVD before the beginning of this study. A total of 11 043 patients with type 2 diabetes were included in the final analysis (Figure 1).

### 2.5 Definition of covariates

Data on covariates were obtained from follow‐up evaluations during the 12‐month BP measurements period. Covariates included the demographic characteristics, lifestyle characteristics, disease information, treatment modalities of the patients, and cumulative mean of SBP and DBP. Demographic characteristics included age, sex, length of education, marital status, and medical insurance. Lifestyle characteristics included smoking status, drinking habit, and exercise status. Current smokers were defined as patients who had smoked within recent 2 weeks. Current alcohol drinkers were defined as individuals who consumed any type of alcohol within the past 2 weeks. Frequent exerciser was defined as performing ≥30 min of physical activities every day. Disease information consisted of the history of CVD and durations of diabetes. Treatment modalities included the use of antihypertensive drugs, oral antidiabetic drugs or insulin, and the use of lipid‐lowering drugs, which were extracted from the last follow‐up record of 12‐month BP assessment period.

### 2.6 Statistical analysis

In the descriptive analysis, data were expressed as means (SD) for continuous variables or as frequencies (percentages) for categorical variables. We used analysis of variance or chi‐square tests to compare the baseline characteristics of participants. The survival and disease‐free probability of primary outcomes according to BPV categories were calculated using Kaplan–Meier curves, and a log‐rank test was performed to explore the differences among the groups. The proportional‐hazards assumption was evaluated using the Schoenfeld residuals test with the logarithm of the cumulative hazards function based on Kaplan–Meier estimates for each BPV category. There was no significant departure from the proportionality hazard assumptions over time. Multivariate Cox proportional hazards regression models were then used to calculate the hazard ratios (HR) and 95% confidence intervals (95% CI) between BPV and macrovascular complications and microvascular complications, where the lowest quartile of BPV was defined as the reference. To control for potential confounding factors, we adjusted for age and sex in the first model. The second model was adjusted for the demographic characteristics, lifestyle characteristics, disease information, treatment modalities of the patients, and cumulative mean of SBP and DBP. Restricted cubic spline curves were performed to visualize the associations between BPV and the risks of macrovascular and microvascular complications with the use of the “Hmisc” packages in the R program (R Foundation for Statistical Computing, Vienna, Austria). We also performed a stratified analysis by age (<65 vs ≥65 years), sex (female vs male), the use of antihypertensive drugs (with vs without), and history of CVD (with vs without). Statistical analyses were performed using SAS version 9.4 (SAS Institute Inc., Cary, NC, USA) and R software version 3.6.3 (http://www.R-project.org). Statistical significance was set at *p* < 0.05.

## 3 RESULTS

### 3.1 Baseline characteristics of the study population

Characteristics of the 11 403 patients with type 2 diabetes included in this study were shown in Table 1. The mean age was 56.52 years, 46.25% were female, and the average duration of diabetes was 5.79 years. The mean BP was 128.12/78.99 mm Hg. Compared with patients in the lowest quartile of SD group (SD of SBP ≤4.27 mm Hg), patients in the highest quartile of SD group (SD of SBP >9.47 mm Hg) were older, had higher body mass index (BMI), and were more likely to receive glucose‐lowering treatment, antihypertensive drugs, and lipid‐lowering drugs.

**TABLE 1:** Baseline characteristics of included patients in this study

	Total	Quintile 1	Quintile 2	Quintile 3	Quintile 4	*p* value
		SD ≤4.27 mm Hg	4.27< SD ≤6.45 mm Hg	6.45< SD ≤9.47 mm Hg	SD >9.47 mm Hg	
	*n* = 11 043	*N* = 2775	*N* = 2748	*N* = 2762	*N* = 2758	
	100%	25.13%	24.88%	25.01%	24.98%	
Social demographics						
Age (years) (mean ± SD)	56.52 ± 10.77	55.94 ± 11.01	55.51 ± 10.64	56.36 ± 10.83	58.28 ± 10.40	<0.0001^a^
Gender, *n* (%)						<0.0001
Male	5936 (53.75)	1509 (54.38)	1586 (57.71)	1515 (54.85)	1326 (48.08)	
Female	5107 (46.25)	1266 (45.62)	1162 (42.29)	1247 (45.15)	1432 (51.92)	
Body mass index, (mean ± SD)	24.30 ± 3.44	24.10 ± 4.09	24.22 ± 3.23	24.32 ± 3.11	24.55 ± 3.22	<0.0001^a^
Length of education, *n* (%)						<0.0001
≥9 years	1043 (9.63)	270 (9.99)	293 (10.84)	255 (9.39)	225 (8.30)	
6 ~ 9 years	6536 (60.34)	1675 (61.99)	1690 (62.52)	1640 (60.41)	1531 (56.45)	
≤6 years	3253 (30.03)	757 (28.02)	720 (26.64)	820 (30.20)	956 (35.25)	
Marriage, *n* (%)						0.3752
Married	10 761 (97.46)	2696 (97.15)	2680 (97.53)	2687 (97.32)	2698 (97.86)	
Single	280 (2.54)	79 (2.85)	68 (2.47)	74 (2.68)	59 (2.14)	
Insurance, *n* (%)						<0.0001
With insurance	4748 (43.40)	1203 (43.97)	1258 (46.18)	1246 (45.57)	1041 (37.91)	
Without insurance	6192 (56.60)	1533 (56.03)	1466 (53.82)	1488 (54.43)	1705 (62.09)	
Lifestyle characteristics						
Smoking status, *n* (%)						0.0308
Current nonsmoker	7505 (85.90)	1767 (87.56)	1857 (86.25)	1899 (84.48)	1982 (85.50)	
Current smoker	1232 (14.10)	251 (12.44)	296 (13.75)	349 (15.52)	336 (14.50)	
Drinking status, *n* (%)						0.0073
Current nondrinker	7989 (90.58)	1886 (92.27)	1972 (90.92)	2023 (89.39)	2108 (89.93)	
Current drinker	831 (9.42)	158 (7.73)	197 (9.08)	240 (10.61)	236 (10.07)	
Physical exercise, *n* (%)						0.0007
Infrequent exerciser	5098 (57.80)	1252 (61.25)	1267 (58.41)	1277 (56.43)	1302 (55.55)	
Frequent exerciser	3722 (42.20)	792 (38.75)	902 (41.59)	986 (43.57)	1042 (44.45)	
Treatment modalities, n (%)						
Any use of blood glucose treatment	9540 (86.39)	2292 (82.59)	2363 (85.99)	2422 (87.69)	2463 (89.30)	<0.0001
Any use of BP lowering drugs	2739 (24.80)	476 (17.15)	555 (20.20)	660 (23.90)	1048 (38.00)	<0.0001
Any use of lipid‐lowering drugs	1553 (14.06)	264 (9.51)	361 (13.14)	429 (15.53)	499 (18.09)	<0.0001
BP during the measurement period, (mean ± SD)						
Mean SBP, mm Hg	128.12 ± 9.20	126.64 ± 7.19	126.34 ± 7.42	127.07 ± 8.71	132.46 ± 11.43	<0.0001^a^
Mean DBP, mm Hg	78.99 ± 5.85	78.30 ± 5.04	78.39 ± 5.27	78.67 ± 5.84	80.60 ± 6.80	<0.0001^a^
Disease characteristics at baseline						
History of cardiovascular disease, *n* (%)	357 (3.23)	79 (2.85)	76 (2.77)	99 (3.58)	103 (3.73)	0.0877
Duration of diabetes (year), (mean ± SD)	5.79 ± 4.71	6.04 ± 4.70	5.69 ± 4.50	5.63 ± 4.64	5.82 ± 4.98	0.0058^a^

Abbreviations: BP, blood pressure; DBP, diastolic blood pressure; SBP, systolic blood pressure.

^a^ This *p* value is associated with analysis of variance; all other *p* values are associated with χ^2^ tests.

### 3.2 Visit‐to‐visit variability of blood pressure and risks of macrovascular and microvascular complications

There were 330 macrovascular complications and 542 microvascular complications. To demonstrate the role of BPV in CVD in more detail, we reported HR and 95% CI for macrovascular and microvascular complications across quartiles of SD‐SBP, SD‐DBP, CV‐SBP, CV‐DBP, ARV‐SBP, and ARV‐DBP in Table 2. The risks of macrovascular and microvascular events were significantly associated with higher SD of SBP and DBP, even after multivariate adjustments for mean BP and other baseline information (*p* for trend <0.05). In contrast to participants with lowest quartile of SD of SBP and DBP, higher risks were observed in patients with the highest quartile of SD of SBP and DBP for macrovascular complications (SD‐SBP: HR = 1.78, 95% CI: 1.24–2.57; SD‐DBP: HR = 2.20, 95% CI: 1.50–3.25) and microvascular complications (SD‐SBP: HR = 1.85, 95% CI: 1.39–2.46; SD‐DBP: HR = 1.82, 95% CI: 1.36–2.44). The CV and ARV of SBP and DBP also had significant associations with macrovascular and microvascular complications.

**TABLE 2:** HRs and 95% CIs for macrovascular and microvascular complications associated with visit‐to‐visit SBP and DBP variability

		Macrovascular complications				Microvascular complications			
		Model 1^a^	*p* for trend	Model 2^b^	*p* for trend	Model 1^a^	*p* for trend	Model 2^b^	*p* for trend
		HR (95% CI)		HR (95% CI)		HR (95% CI)		HR (95% CI)	
SD of SBP, mm Hg			<0.0001		0.0136		<0.0001		<0.0001
Quintile 1	SD ≤4.27	1.00 (reference)		1.00 (reference)		1.00 (reference)		1.00 (reference)	
Quintile 2	4.27 < SD≤6.45	1.11 (0.80–1.53)		1.28 (0.88–1.87)		0.94 (0.72–1.24)		1.06 (0.77–1.46)	
Quintile 3	6.45 < SD≤9.47	1.69 (1.25–2.29)		1.52 (1.07–2.16)		1.67 (1.31–2.13)		1.73 (1.30–2.30)	
Quintile 4	SD >9.47	2.25 (1.68–3.02)		1.78 (1.24–2.57)		2.68 (2.15–3.35)		1.85 (1.39–2.46)	
SD of DBP, mm Hg			<0.0001		0.0002		<0.0001		0.0008
Quintile 1	SD ≤3.29	1.00(reference)		1.00(reference)		1.00(reference)		1.00(reference)	
Quintile 2	3.29 < SD≤4.81	1.53 (1.13–2.08)		1.32 (0.92–1.90)		1.61 (1.25–2.06)		1.45 (1.08–1.93)	
Quintile 3	4.81 < SD≤6.70	1.77 (1.31–2.41)		1.83 (1.29–2.60)		2.03 (1.59–2.60)		1.52 (1.14–2.02)	
Quintile 4	SD > 6.70	2.49 (1.82–3.42)		2.20 (1.50–3.25)		2.65 (2.09–3.37)		1.82 (1.36–2.44)	
CV of SBP			<0.0001		0.0143		<0.0001		<0.0001
Quintile 1	CV ≤3.37	1.00(reference)		1.00(reference)		1.00(reference)		1.00(reference)	
Quintile 2	3.37 < CV ≤5.12	1.29 (0.95–1.77)		1.40 (0.97–2.02)		0.98 (0.75–1.28)		1.09 (0.80–1.49)	
Quintile 3	5.12 < CV ≤7.39	1.64 (1.20–2.23)		1.47 (1.04–2.09)		1.74 (1.36–2.21)		1.74 (1.31–2.31)	
Quintile 4	CV >7.39	2.32 (1.72–3.14)		1.8 0 (1.25–2.60)		2.71 (2.17–3.40)		1.89 (1.43–2.50)	
CV of DBP			<0.0001		0.0008		<0.0001		<0.001
Quintile 1	CV ≤4.20	1.00(reference)		1.00(reference)		1.00(reference)		1.00(reference)	
Quintile 2	4.20 < CV≤6.12	1.37 (1.02–1.85)		1.27 (0.89–1.82)		1.62 (1.26–2.07)		1.53 (1.15–2.02)	
Quintile 3	6.12 < CV≤8.49	1.77 (1.31–2.40)		1.83 (1.29–2.59)		1.95 (1.52–2.50)		1.44 (1.08–1.92)	
Quintile 4	CV >8.49	2.29 (1.67–3.15)		2.01 (1.36–2.98)		2.84 (2.24–3.61)		2.03 (1.52–2.70)	
ARV of SBP			<0.0001		0.0007		<0.0001		<0.0001
Quintile 1	ARV≤4.00	1.00(reference)		1.00(reference)		1.00(reference)		1.00(reference)	
Quintile 2	4.00 < ARV≤6.00	1.58 (1.17–2.14)		1.49 (1.06–2.09)		1.42 (1.10–1.82)		1.44 (1.08–1.92)	
Quintile 3	6.00 < ARV≤9.00	1.93 (1.42–2.64)		1.46 (1.02–2.10)		1.86 (1.43–2.42)		1.69 (1.24–2.29)	
Quintile 4	ARV >9.00	2.86 (2.08–3.94)		2.28 (1.54–3.39)		3.25 (2.57–4.10)		2.18 (1.64–2.91)	
ARV of DBP			<0.0001		0.0016		<0.0001		<0.0001
Quintile 1	ARV ≤ 3.00	1.00(reference)		1.00(reference)		1.00(reference)		1.00(reference)	
Quintile 2	3.00 < ARV≤4.67	1.29 (0.96–1.71)		1.53 (1.09–2.16)		1.35 (1.05–1.73)		1.40 (1.05–1.87)	
Quintile 3	4.67 < ARV ≤ 6.67	1.86 (1.34–2.58)		1.50 (1.00–2.26)		2.34 (1.81–3.04)		1.78 (1.31–2.41)	
Quintile 4	ARV > 6.67	2.29 (1.67–3.14)		2.13 (1.45–3.12)		3.25 (2.53–4.17)		2.23 (1.66–3.00)	

Abbreviations: ARV, absolute real variability; CI, confidence interval; CV, coefficient of variation; DBP, diastolic blood pressure; HR, hazard ratio; SBP, systolic blood pressure.

^a^ Model 1 adjusted for age, sex.

^b^ Model 2 adjusted for age, sex, body mass index, education, marriage, insurance, smoke, drink, exercise, use of blood glucose drugs, use of blood pressure lowering drugs, use of lipid‐lowering drugs, duration of diabetes, and mean SBP and DBP.

**FIGURE 2:**
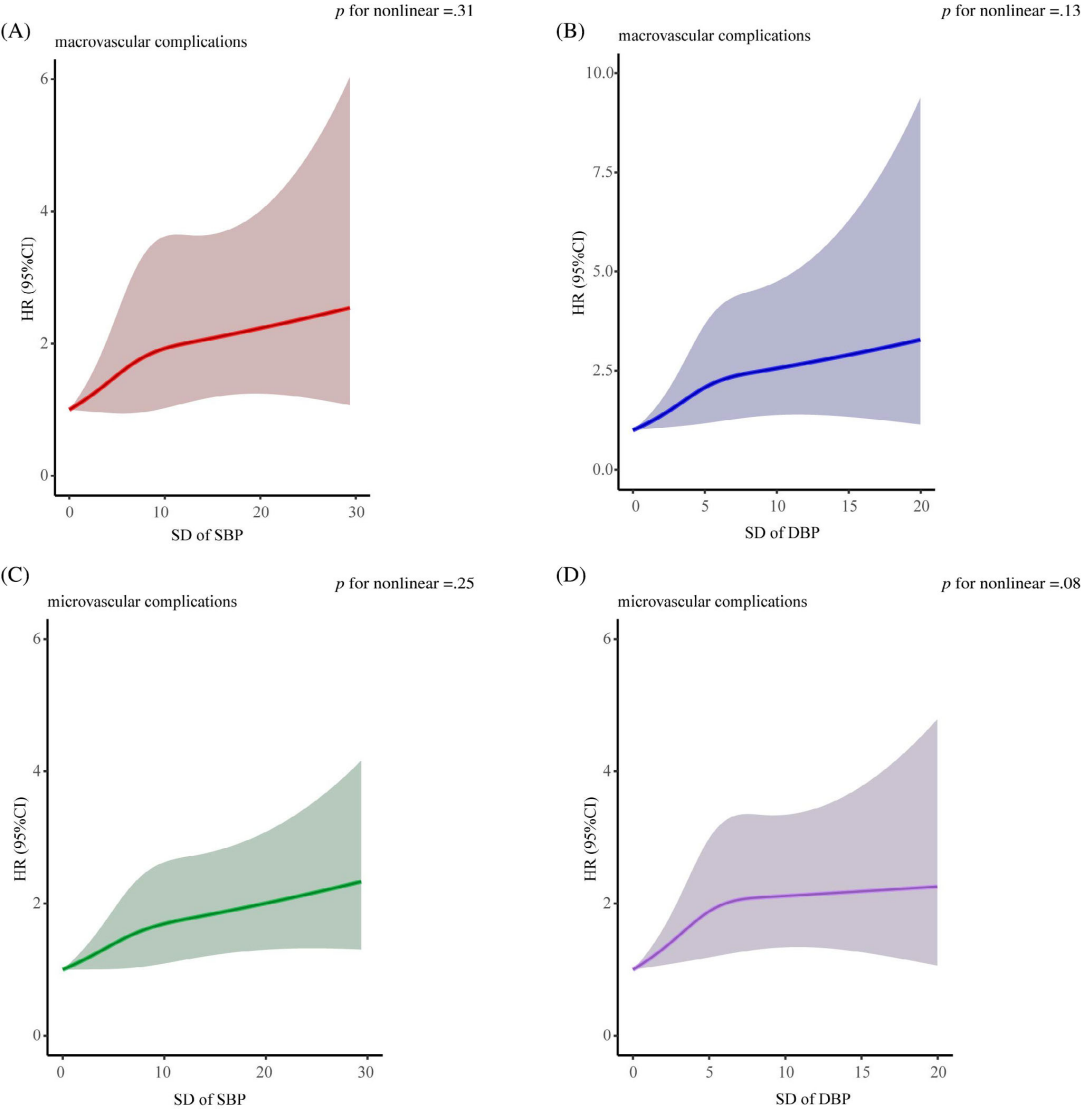
Associations between visit‐to‐visit variability (VVV) of blood pressure (BP) and microvascular and macrovascular complications in patients with type 2 diabetes. The SD of BP was assessed as a continuous variable using restricted cubic spline regression with three knots, adjusted for age, sex, body mass index, education, marriage, insurance, smoke, drink, exercise, use of blood glucose treatment, BP‐lowering drugs, lipid‐lowering drugs, mean systolic blood pressure (SBP) and diastolic blood pressure (DBP), history of cardiovascular disease, and duration of diabetes. Splines and 95% confidence intervals (CI) were shown. Shaded regions showed 95% CI limits. The *x* axis represented SD of BP in mm Hg, and the *y* axis represents the risks of macrovascular complications and microvascular complications. (A) for VVV of SBP and macrovascular complication. (B) for VVV of DBP and macrovascular complication. (C) for VVV of SBP and microvascular complications. (D) for VVV of DBP and microvascular complications. HR, hazard ratio.

We further evaluated the relationships between BPV and macrovascular and microvascular events using a restricted cubic spline curve with SD of SBP and DBP as a continuous variable. Consistent with the results in Table 2, the risks of macrovascular and microvascular complications were higher with the increase of SD of SBP and DBP (Figure 2). Moreover, we found that linear associations between the BPV and macrovascular and microvascular complications may exist (*p* for nonlinear >0.05).

### 3.3 Subgroup analysis

**FIGURE 3:**
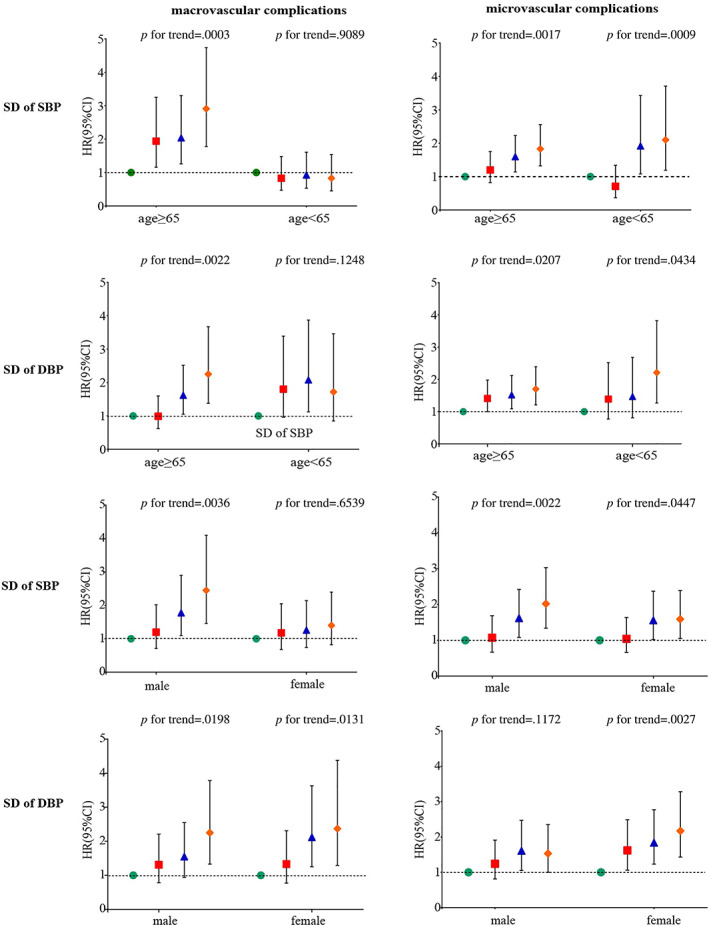
Associations between visit‐to‐visit variability (VVV) of blood pressure and microvascular and macrovascular complications in patients with type 2 diabetes, stratified by age (<65 vs ≥65 years), sex (female vs male). CI, confidence interval; DBP, diastolic blood pressure; HR, hazard ratio; SBP, systolic blood pressure.

**FIGURE 4:**
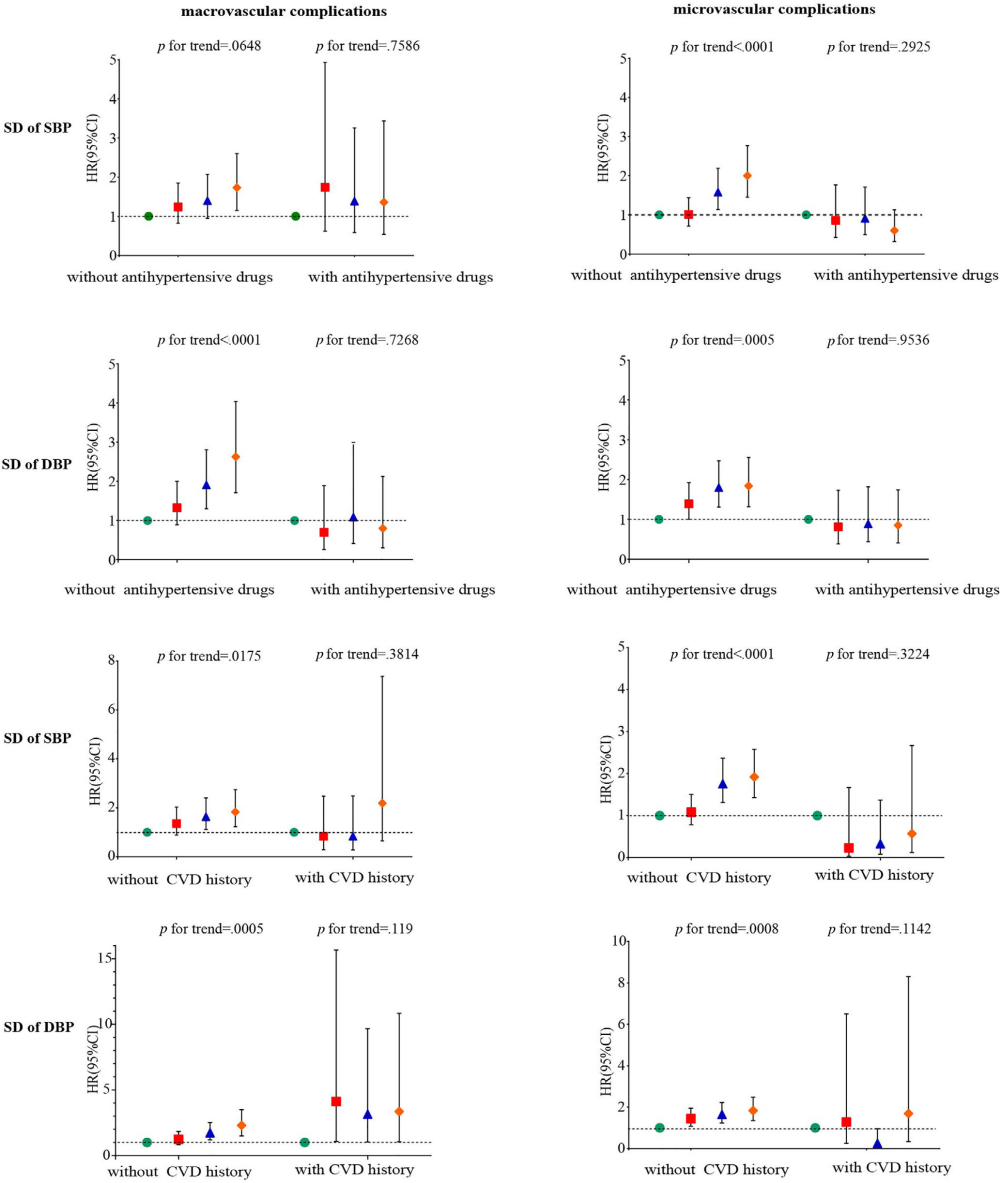
Associations between visit‐to‐visit variability (VVV) of blood pressure and risks of microvascular and macrovascular complications in patients, stratified by the use of antihypertensive drugs (with vs without) and history of CVD (with vs without). CI, confidence interval; CVD, cardiovascular disease; DBP, diastolic blood pressure; HR, hazard ratio; SBP, systolic blood pressure.

We performed stratified analysis by age (<65 vs ≥65 years), sex (female vs male), the use of anti‐hypertensive drugs (with vs without), and history of CVD (with vs without). Among participants over 65, with the increase of the VVV of SBP and DBP, the risks of macrovascular and microvascular events were higher, and the trend was statistically significant (*p* for trend <0.05). Only significant associations between BPV and microvascular events in patients under 65 years were observed (Figure 3). In participants without history of CVD, those with higher BPV had a higher risk of developing CVD (*p* for trend <0.05). However, higher BPV had no significant increase in risk for CVD in patients with history of CVD (Figure 4). Among patients without use of antihypertensive drugs, higher BPV was a risk factor for CVD (*p* for trend <0.05). However, no significant associations were observed among patients with use of antihypertensive drugs (Figure 4).

## 4 DISCUSSION

Based on the HMPD of primary healthcare institutions in China, we explored the associations between BPV and macrovascular and microvascular complications among patients with type 2 diabetes in a real‐world medical setting. We found that BPV was an independent risk factor for CVD and our findings demonstrated a direct linear relationship between BPV and CVD. Moreover, the results also identified that the optimal BPV target: SD of SBP <6.45 mm Hg and SD of DBP <4.81 mm Hg.

Previous studies advocated that the optimal target SD of SBP should be <10 mm Hg to protect cardiovascular health in patients with diabetes.^19^, ^20^ In this study, the target of BPV seems to be more stringent. The main reasons are as follows: previous studies mainly focused on macrovascular complications, and the targets of BPV in these studies were only for macrovascular events. The objective of BPV proposed by this research integrated macrovascular and microvascular events, and microvascular events were more sensitive to BPV in our study. Therefore, the targets of BPV formed by us were more restrictive.

Several mechanisms have been put forward to account for the association between BPV and adverse cardiovascular outcomes. First, the elevation in the BPV is positively associated with adverse alterations in carotid intima‐media thickness,^21^ which may be a marker of atherosclerosis^22^ and thus could promote CVD. Second, long‐term BPV is associated with endothelial dysfunction and inflammation,^23^, ^24^ which may result in adverse effects on cardiovascular health.^25^, ^26^ Third, BPV may have harmful effects to myocardial ischemia. As coronary blood flow peaks during diastole, repeated transient declines in DBP over time may put cardiac tissue at increased risks of relative hypoperfusion.^14^

We found that the risk of CVD due to BPV was higher in patients over 65 years. However, the complex relationships were still unclear. Aging is accompanied by deterioration of cardiovascular homeostasis and metabolic disturbances, which are highly interconnected adverse vascular and cardiac phenotypes responsible for myocardial infarction, stroke, and heart failure.^27^ In participants without history of CVD, higher BPV was a risk factor for developing CVD. However, no significant associations were observed among patients with CVD history. The lack of significant association might be because of the low proportion of patients with CVD history participating in this study (3.23%), giving insufficient power to detect meaningful differences.^28^ Among the patients with the use of antihypertensive drugs, no significant associations between BPV and CVD were found. The possible reason was that antihypertensive drugs were used to lower BP levels, which was considered a risk factor for CVD. And substantial evidence also concluded that pharmacological blood pressure lowering was effective for prevention of cardiovascular events.^29^, ^30^

Our study was one of the few studies conducted on BPV and CVD among Chinese patients with type 2 diabetes. Meanwhile, we acknowledged several limitations to our study. First, with a mean follow‐up of only 43.57 months, it is possible that we observed insufficient outcomes. Therefore, we might have underestimated the incidence of CVD. Second, selection of study participants who had received 12‐month follow‐up evaluation might be a source of selection bias because patients with insurance (43.40% vs 36.97%, *p* < 0.05) were more likely to participate in the regular health service. Third, our study did not cover patients without diabetes. Therefore, we could not perform analysis among non‐diabetic individuals and explore the difference on cardiovascular outcomes caused by BPV between diabetic and nondiabetic individuals. Finally, we did not consider whether the BPV could change during the follow‐up period, which might have potential effects on the results.

## 5 CONCLUSIONS

In conclusion, we explored the effect of BPV on the risk of CVD among patients with type 2 diabetes and found that VVV of SBP and DBP may be potential predictors for the development of macrovascular and microvascular complications in patients with type 2 diabetes. In addition to monitoring BP targets for patients with type 2 diabetes, clinical professionals should also remain vigilant about the visit‐to‐visit fluctuation of BP.

## DISCLOSURE

No conflicts of interest in this work.
